# Green Revolution Dwarfing Alleles Are Rare in Spring Bread Wheat Germplasm Grown in Russia

**DOI:** 10.3390/plants15213270

**Published:** 2026-10-23

**Authors:** Boburjon B. Najodov, Anastasiya G. Chernook, Alexey I. Morgounov, Timofey D. Mokhov, Anastasiya A. Zolotko, Svetlana S. Bazhenova, Nataliya V. Davydova, Maria E. Mukhordova, Igor A. Belan, Lyudmila P. Rosseeva, Vladimir P. Shamanin, Valentina S. Rubets, Gennady I. Karlov, Mikhail G. Divashuk

**Affiliations:** 1 All-Russian Research Institute of Agricultural Biotechnology, 42 Timiryazevskaya Street, Moscow 127550, Russiaalexey.morgounov@gmail.com (A.I.M.); divashuk@gmail.com (M.G.D.); 2 Russian State Agrarian University—Moscow Timiryazev Agricultural Academy, 49 Timiryazevskaya Street, Moscow 127550, Russia; 3 Federal Research Center “Nemchinovka”, 6 Agrokhimikov Street, Novoivanovskoe, Odintsovo 143026, Moscow Region, Russia; 4 Omsk Agricultural Scientific Center, 26 Korolev Avenue, Omsk 644012, Russia; 5 Faculty of Agrotechnology, Omsk State Agrarian University, 1 Institutskaya Square, Omsk 644008, Russia; 6 N.V. Tsitsin Main Botanical Garden of the Russian Academy of Sciences, 4 Botanicheskaya Street, Moscow 127276, Russia

**Keywords:** spring bread wheat, Green Revolution, *Rht-B1b*, *Rht-D1b*, dwarfing genes, reduced height, marker-assisted selection

## Abstract

The Green Revolution dwarfing alleles *Rht-B1b* and *Rht-D1b* shorten the stem and reduce lodging, and under intensive management often raise yield, although this advantage may disappear in dry or low-input environments. Their frequency in spring bread wheat grown in Russia has not been established. We genotyped 370 accessions. None carried *Rht-B1e* or *Rht-B1p*, only eight carried *Rht-B1b* (2.2%) and one, Zolushka, carried *Rht-D1b* (0.3%); two were heterogeneous at *Rht-B1*. These figures describe this collection, not a probability sample of Russian spring bread wheat. Pedigrees trace the eight *Rht-B1b* carriers to five sources: three inherited the allele from a winter bread wheat parent, two from CIMMYT semi-dwarf material, and three belong to a single introduced lineage; none derives it from Russian spring wheat breeding. In 43 accessions grown in Moscow over three seasons, the three *Rht-B1b* carriers were 15.4% shorter than the 39 wild-type accessions (60.7 versus 71.8 cm, *p* = 0.021), whereas spike traits and grain yield in Moscow and Omsk did not differ significantly; with three carriers these comparisons are exploratory and small effects cannot be excluded. Because one field-evaluated carrier was as tall as the wild type, marker-based identification is needed wherever these alleles are deployed.

## 1. Introduction

Reduced-height genes have played a central role in improving wheat productivity since the Green Revolution of the 1960s and 1970s. The introduction of semi-dwarf cultivars carrying mutant alleles of *Rht* genes, together with increased fertiliser input and intensive agronomic practices, contributed to higher grain yield, improved lodging resistance, and greater stability of wheat production [1,2,3]. Among these genes, the classical gibberellin-insensitive dwarfing alleles *Rht-B1b* (=*Rht1*) and *Rht-D1b* (=*Rht2*) became the most widely used Green Revolution alleles and remain important genetic factors in modern wheat breeding [1,4,5].

Both are mutant variants of genes encoding DELLA proteins of the gibberellin signalling pathway, and the resulting gibberellin-insensitive phenotype is characterised by suppressed internode elongation, reduced plant height, and improved lodging resistance [1,4,6,7,8]. At the protein level, the semi-dwarfing effect of these alleles results from N-terminally truncated RHT-1 proteins generated by translational reinitiation, which escape gibberellin-induced degradation [9]. Under intensive cropping systems, where high nitrogen input and favourable moisture increase lodging risk, these alleles provide a clear agronomic advantage [3,10,11]. Their effects, however, extend beyond plant height to coleoptile length, seedling vigour, vegetative biomass, spike traits, nitrogen use efficiency and grain quality, so that their breeding value depends strongly on genetic background, environment and the level of agronomic input, and excessive height reduction may be disadvantageous under drought-prone or low-input conditions [12,13,14,15,16,17,18].

This context-dependence is reflected in the contrasting use of dwarfing alleles in the two wheat forms grown in Russia. In winter bread wheat breeding, reduced-height genes have been widely incorporated into high-yielding cultivars developed for intensive production systems [19,20,21]. In spring bread wheat, by contrast, the occurrence of *Rht-B1b* and *Rht-D1b* appears to be considerably more limited [5,22,23]. This raises the question addressed in the present work: why are dwarfing alleles of global importance, successfully deployed in many breeding programmes worldwide, still poorly represented in spring bread wheat breeding material?

Answering this question requires knowing how frequent these alleles are. As of 2026, 362 spring bread wheat cultivars are listed in the State Register of Breeding Achievements Approved for Use in the Russian Federation, reflecting the high varietal diversity of this crop and its major role in Russian agriculture [24]. Despite this diversity, the distribution of *Rht-B1b* and *Rht-D1b* in Russian spring bread wheat has not been comprehensively characterised, unlike that in winter bread wheat and durum wheat germplasm collections. Large-scale molecular analysis of the *Rht-B1* and *Rht-D1* loci is therefore needed to determine the extent to which the Green Revolution dwarfing alleles have been incorporated into spring bread wheat germplasm, and marker-based identification is needed to recognise the rare carriers that phenotypic selection alone would not reliably detect [4,22,25].

To address this gap, we analysed 370 unique spring bread wheat genotypes of different breeding origin, including registered cultivars and breeding lines, and additionally evaluated a subset of 43 accessions in two contrasting environments. The objectives were (i) to screen the collection with allele-specific markers for the gibberellin-insensitive dwarfing alleles *Rht-B1b* and *Rht-D1b*; (ii) to estimate the frequency of *Rht-B1b* and *Rht-D1b* in the collection and to trace the breeding origin of the carriers identified; and (iii) to assess the phenotypic expression and agronomic performance of these alleles in the field-evaluated subset. To our knowledge, this is the largest survey of *Rht-B1b* and *Rht-D1b* in Russian spring bread wheat to date, extending our earlier analysis of 71 accessions [23]. The study was designed as a survey of allele frequency and pedigree origin in existing germplasm; the field data describe the agronomic behaviour of the carriers that were available and do not estimate allele effects, which would require near-isogenic lines or biparental populations. The Results and Discussion address these three questions in turn: how frequent the alleles are, where the carriers obtained them, and how the carriers behave in the field.

## 2. Materials and Methods

### 2.1. Plant Material

The 370 accessions were selected to represent the major breeding programmes of Russia and Kazakhstan and international centres (CIMMYT). About 170 of them are cultivars entered in the State Register of Breeding Achievements Approved for Use in the Russian Federation [24]; the remainder are advanced breeding lines from current breeding programmes, entries of competitive variety trials, and other accessions maintained in the collections named below. The collection comprises material from 39 breeding programmes. Of the 370 accessions, 316 originate from Russia, 21 from Kazakhstan, 20 from CIMMYT (Mexico), 12 from western and central Europe (Germany, Poland, France, Austria, the Czech Republic and Portugal) and one from Belarus. The contribution of the individual programmes is uneven: 142 accessions were developed at the Omsk Agricultural Scientific Center and 40 at Omsk State Agrarian University, so that 182 accessions, or 49% of the collection, originate from two institutions in a single region; a further 69 come from the Federal Research Center “Nemchinovka” (Novoivanovskoe, Moscow Region, Russia) whereas 13 programmes are represented by a single accession each. The collection reflects the material that was available to us and is not a probability sample of the spring bread wheat grown in Russia, so the allele frequencies reported below apply to this collection. Seed of all accessions was obtained from the originating breeding programmes and from the working collection of the Department of Genetics, Breeding and Seed Production of the Russian State Agrarian University—Moscow Timiryazev Agricultural Academy (Moscow, Russia). The 43 accessions used for field evaluation were selected to represent the different breeding origins of the collection, before the genotyping results were available; they include three of the eight *Rht-B1b* carriers (Granny, Granova and line No. 153) and the single *Rht-D1b* carrier (Supplementary Table S1). All 370 accessions were genotyped (Supplementary Table S2); the 43 were evaluated in Moscow and, together with 18 further accessions, in Omsk (Supplementary Table S3). The State Register [24] was used as a documentary source only, for registration records and pedigrees (including that of Granny [26]), for the official variety-testing yields given in Supplementary Table S3 and for the descriptions of lodging resistance quoted in Section 4.4.

### 2.2. Field Trials

The Moscow trial comprised 43 accessions evaluated over three seasons (2022–2024) at the Field Experiment Station of the Russian State Agrarian University—Moscow Timiryazev Agricultural Academy (Moscow, Russia). The soil is soddy-podzolic, loamy, with a humus content of 2.4–2.5% and pH 5.8–6.2. The preceding crop was bare fallow. Pre-sowing fertiliser was applied at N_60_P_60_K_60_; no additional nitrogen was applied during the growing season. At full ripeness, 10 plants were sampled at random from each plot, giving a nominal 30 plants per accession per year; the number actually recorded ranged from 29 to 31 per accession and year, except for the standard cultivar Zlata, which occupied three plots in each replicate and is represented by 90 plants per year, and for line No. 35 in 2024 (19 plants). The three seasons together yielded 4038 individual plant records. The following traits were recorded on each plant: plant height, main stem height, total and fertile spikelet number, spike length, number of grains per spike, and grain weight per spike. Phenological stages were recorded per plot. Grain yield was determined per plot and expressed in t ha^−1^. Grain protein content (%), gluten content (%) and vitreousness (%) were determined for each accession and year on whole grain by Fourier-transform near-infrared spectroscopy with an InfraLUM FT-12 analyser (Lumex, St. Petersburg, Russia), using the manufacturer’s calibration models for bread wheat. Weather conditions were recorded at the V.A. Mikhelson Meteorological Observatory (Moscow) and are summarised in Supplementary Table S4. Agronomic practices followed the standards for the zone. Sowing was carried out with an SKS-6 plot drill (Russia) at a rate of 540 viable seeds per m^2^ (5.4 million per hectare), in three replicates; the harvest plot area was 1 m^2^. The trial was established and conducted according to Dospekhov [27] as a design with a repeated standard: the three replicates were placed at random in the field, and within each replicate, without randomisation, the 42 other accessions were sown in the same fixed order, and the standard cultivar Zlata, one of the 43 accessions, occupied every fifteenth plot (plots 8, 23 and 38), giving 45 plots per replicate and 135 plots per season. Lodging resistance, phenology and thousand-grain weight were assessed according to the Methods of State Variety Trials of Agricultural Crops [28], lodging on a five-point scale from 5 (no lodging) to 1 (nearly all plants lodged); lodging was not scored in the Omsk trial. Sheaves were threshed with an MPSU-500 plot thresher and analysed in the laboratory of the Department of Genetics, Breeding and Seed Production of the same university.

The Omsk trial was conducted in 2025 at the experimental field of the P.A. Stolypin Omsk State Agrarian University (55°02′ N, 73°31′ E), in the southern forest-steppe subzone of Western Siberia, Omsk region, Russia. It comprised 61 accessions, including the 43 from the Moscow trial; the allelic-group comparison in Section 3.4 uses all 61. The preceding crop was bare fallow. Accessions were sown at a rate of 5.4 million viable seeds per hectare on plots of 1 m^2^ in three replicates. Grain yield was determined per plot. An overall breeding score was additionally assigned to each accession by the Omsk breeders on a five-point scale, integrating plant habit, lodging resistance, maturity and grain appearance; the score is a subjective expert assessment and is not independent of plant height. Because the two locations were tested in different years, the Omsk trial is presented as an independent verification rather than as a second environment of a multi-location design.

### 2.3. DNA Extraction and Genotyping

Genomic DNA was isolated from dried leaves of adult plants, separately from two individual plants and from a bulk of eight plants per accession, using the standard CTAB method [29]. DNA quality and concentration were assessed with a NanoDrop spectrophotometer (Thermo Fisher Scientific, Waltham, MA, USA). The dwarfing alleles *Rht-B1b* and *Rht-D1b* were identified with KASP assays (LGC Genomics, Hoddesdon, UK) targeting the causal polymorphisms originally described for these alleles [4] and using the assay designs validated for bread wheat by Rasheed et al. [25]. The collection was additionally screened for the gibberellin-insensitive alleles *Rht-B1e* (=*Rht11*) and *Rht-B1p* (=*Rht17*) with the allele-specific markers described by Li et al. [30] and Bazhenov et al. [31], respectively. Accessions designated *Rht-B1a* (wild type) in this paper therefore carry none of *Rht-B1b*, *Rht-B1e* and *Rht-B1p*, and accessions designated *Rht-D1a* do not carry *Rht-D1b*; other, rarer alleles at the two loci were not assayed. Amplification was performed on a CFX96 Touch Real-Time PCR Detection System and analysed with Bio-Rad CFX Manager software version X.X (Bio-Rad Laboratories, Hercules, CA, USA). Control cultivars with known allelic states were included: Altigo, carrying *Rht-B1a*/*Rht-D1b*, and Vassa, carrying *Rht-B1b*/*Rht-D1a*. The genotypes of the eight *Rht-B1b* carriers and of the *Rht-D1b* carrier were confirmed by repeated genotyping. Where both alleles were detected among the samples of an accession, the accession was scored as heterogeneous at that locus; such accessions were excluded from the allelic-group comparisons. Genotyping was performed at the Laboratory of Applied Genomics and Crop Breeding of the All-Russian Research Institute of Agricultural Biotechnology.

### 2.4. Statistical Analysis

Because the dwarfing alleles were represented by a small number of accessions, the accession mean was used as the unit of replication in all comparisons between allelic groups, both for the overall means and for the individual seasons. Differences between accessions carrying *Rht-B1b* and accessions carrying the wild-type alleles were tested with the two-sided Mann–Whitney *U* test, with exact *p* values obtained from the permutation distribution of ranks, which accounts for tied values, and differences with *p* < 0.05 were considered significant. Because the Moscow plots were not randomised, plant height was also adjusted for position in the field with the repeated standard: each plant value was multiplied by the ratio of the seasonal mean height of Zlata to the height of Zlata interpolated linearly between the two nearest standard plots of the same replicate, and the allelic groups were compared again on the adjusted accession means. For each comparison, the Hodges–Lehmann estimate of the difference between the groups and its 95% confidence interval were also calculated, together with the smallest difference detectable with 80% power at α = 0.05 (Supplementary Table S5); confidence intervals are reported instead of retrospective power, which adds nothing to the *p* value [32]. Figures showing individual plant records are illustrative only. The *Rht-D1b* group comprised a single accession (Zolushka) and was therefore described but not tested statistically; Zolushka was not pooled with the wild-type group in any comparison, so the wild-type group comprises 39 accessions in the Moscow trial, 57 in the Omsk trial and 19 among the registered cultivars with State Register yield data. Records with values outside the biologically plausible range (plant height < 30 cm, spike length < 3 cm, or grain weight per spike > 5 g) were treated as data-entry errors and excluded prior to analysis; the ten excluded records are listed in Supplementary Table S6. Least significant differences (LSD_0.05_) for the Moscow trial, computed from a genotype × replicate analysis of variance on replicate means, are given in Supplementary Table S7; because the plots were not randomised, these values are indicative. Analyses were performed in Python 3 using the pandas, NumPy and SciPy libraries. Given the exploratory nature of these comparisons and the small number of carriers, no correction for multiple testing was applied, and the reported *p* values are to be read as descriptive, not confirmatory.

Generative artificial intelligence was used during the preparation of this manuscript. Claude (Opus 4.5 and Opus 5, Anthropic) was used to verify the summary statistics reported here against the primary field and laboratory records, to check the reference list for completeness and citation order, to translate accession pedigrees from Russian into English for Supplementary Table S1, and to draft and edit passages of the text. DeepSeek-V3 and DeepSeek-R1 were used for language editing and for drafting passages of the text. All original data and all calculations are the authors’ own, and no data were generated or imputed by any of these tools: every value reported here derives from the primary records. The authors have reviewed and edited all output and take full responsibility for the content of this publication.

## 3. Results

### 3.1. Occurrence of Gibberellin-Insensitive Dwarfing Alleles at the Rht-B1 and Rht-D1 Loci

**Figure 1:**
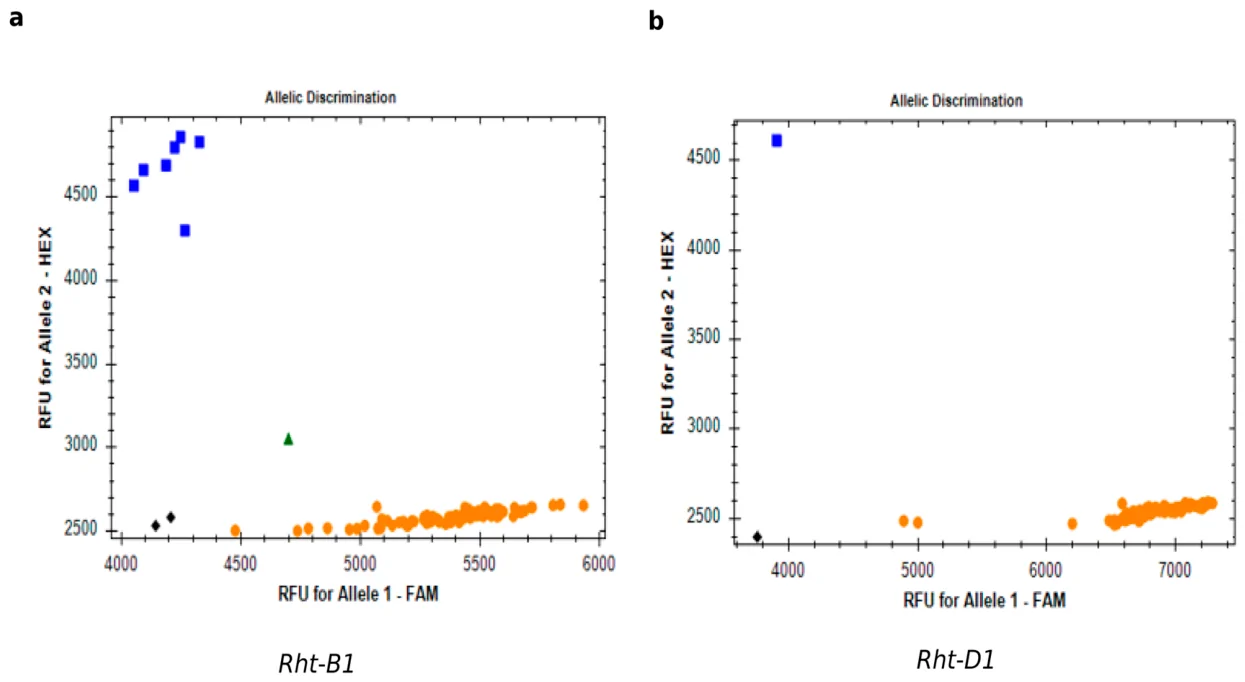
KASP allelic discrimination plots for (**a**) *Rht-B1* and (**b**) *Rht-D1*, as produced by Bio-Rad CFX Manager (x axis, FAM fluorescence for allele 1; y axis, HEX fluorescence for allele 2). Orange circles, samples carrying the wild-type alleles *Rht-B1a* and *Rht-D1a*; blue squares, samples carrying the dwarfing alleles *Rht-B1b* and *Rht-D1b*; green triangle in (**a**), a sample in which both alleles were detected; black diamonds, no-template (water) controls, which define the background fluorescence in each channel. A representative plate is shown.

**Figure 2:**
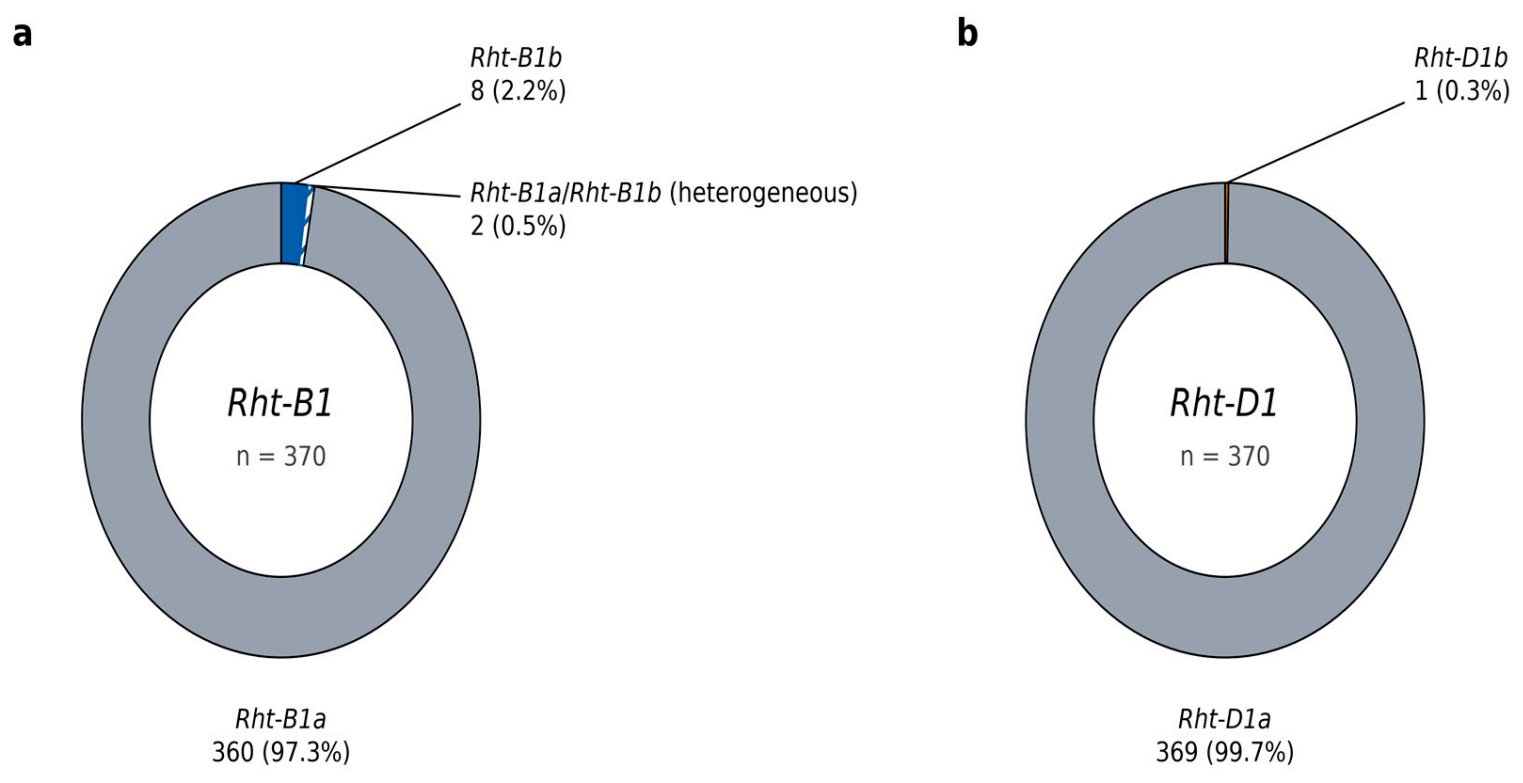
Allelic composition at the (**a**) *Rht-B1* and (**b**) *Rht-D1* loci in the 370 spring bread wheat accessions. Grey, wild-type alleles *Rht-B1a* and *Rht-D1a*; blue, *Rht-B1b*; hatched, accessions heterogeneous at *Rht-B1*, in which both *Rht-B1a* and *Rht-B1b* were detected; orange, *Rht-D1b*. The same colours are used in all figures.

Molecular genetic analysis of the *Rht-B1* and *Rht-D1* loci showed that the wild-type alleles *Rht-B1a* and *Rht-D1a* strongly predominated in the studied spring bread wheat genotypes. Among 370 unique genotypes, *Rht-B1a* was detected in 360 genotypes, whereas the dwarfing allele *Rht-B1b* was detected in only eight; two genotypes showed a heterogeneous allelic state at this locus. At the *Rht-D1* locus, the wild-type allele *Rht-D1a* was detected in 369 genotypes, while the dwarfing allele *Rht-D1b* was identified in a single genotype. None of the 370 accessions carried *Rht-B1e* or *Rht-B1p*. The frequency of *Rht-B1b* was 2.2% counting the eight uniform carriers, or 2.7% if the two heterogeneous accessions are included, and that of *Rht-D1b* was only 0.3% (Figure 1 and Figure 2). These values indicate a very limited incorporation of the classical gibberellin-insensitive dwarfing alleles into the studied germplasm.

### 3.2. Breeding Origin of the Accessions Carrying Dwarfing Alleles

Pedigree analysis showed that the eight *Rht-B1b* carriers do not represent eight independent occurrences of the allele (Table 1; Supplementary Table S1). Three belong to a single lineage: Granova was selected from Granny, and Pamyati Konovalova from the cross (Laval 19 × Granny) × Granny. Granny itself was developed at Saatbau Linz (Austria) from the cross U23 × U513; the State Register does not state the growth habit of the second parent [26], whereas the accession record in our collection describes it as a winter wheat line (Supplementary Table S1). We therefore treat this growth habit as undetermined; if the winter habit is confirmed, six of the eight carriers would have acquired the allele from a winter parent.

The single *Rht-D1b* carrier, Zolushka, has no documented pedigree. It was obtained from the collection of the N.I. Vavilov All-Russian Institute of Plant Genetic Resources (VIR) through the Federal Scientific Center of Legumes and Groat Crops, where it gave the lowest grain yield among eleven accessions in a variety trial in the Orel region in 2019–2020 (2.74 t ha^−1^ against a trial mean of 3.82 t ha^−1^; LSD_0.05_ = 0.4 t ha^−1^) [33]. Granova and Pamyati Konovalova have been evaluated for spike productivity at the same centre [34].

The two Nemchinovka carriers, CVT-87-23 and CVT-89-23, are half-sibs: both derive from line 5662-13 (Yunona × Zlata), crossed with Dandy and with the winter cultivar Kolo, respectively (Table 1). Yunona is a winter bread wheat cultivar from Krasnodar reported to carry *Rht-B1b* [20], and Zlata, genotyped here, carries the wild-type alleles, so Yunona is the most probable donor; in CVT-89-23 Kolo is an alternative winter source, and in CVT-87-23 a contribution from the untested parent Dandy cannot be excluded. In an earlier survey of 71 accessions from the same programme, these two lines were likewise the only carriers of *Rht-B1b* and were 80 cm tall with high lodging resistance [23].

Two further Nemchinovka accessions, CVT-102-23 and CVT-123-23, were heterogeneous at *Rht-B1*; in a self-pollinating species this most likely reflects residual within-accession heterogeneity; both were scored as *Rht-B1a* in the earlier survey of a smaller plant sample [23]. All their recorded parents are of spring habit, and those genotyped here (Agata, Liza and Ester) carry *Rht-B1a*; the parents Kurier and Moskovka were not available, so the origin of the *Rht-B1b* fraction remains unresolved.

The breeding line Lutescens 21/12-1-2 (Omsk Agricultural Scientific Center) was obtained from the cross Omskaya 36 × Yuka. Yuka is a winter bread wheat cultivar reported to carry *Rht-B1b* [20], and Omskaya 36, genotyped here, carries the wild-type alleles, so this is the only carrier for which the donor is established from genotypic rather than documentary evidence. It is also the only carrier among the 182 Omsk-bred accessions of the collection (142 from the Omsk Agricultural Scientific Center and 40 from Omsk State Agrarian University); the cultivar Omskaya 24, whose pedigree also includes a winter cultivar, Krasnodarskaya 39, carries the wild-type alleles (Supplementary Table S1). The line is only moderately short-strawed (83 cm, 4 cm below the standard cultivar Duet) [35]. The two remaining carriers trace to CIMMYT germplasm. The male parent of line No. 153 is a CIMMYT line whose pedigree includes the semi-dwarfs Tobari 66, Ciano 67, Veery #5 and Kauz, the last shown by allele-specific PCR to carry *Rht-B1b* [36]; the pedigree of the Portuguese cultivar Roxo includes the CIMMYT semi-dwarfs Yécora 70 and Bobwhite (Supplementary Table S1). Because *Rht-B1b* is effectively fixed in CIMMYT elite spring wheat [37], CIMMYT ancestry is the most parsimonious reading of both pedigrees, although no ancestor was available for genotyping.

These pedigrees show that the already low frequency of *Rht-B1b* overstates the extent to which the allele has been adopted. The eight carriers trace to five sources: the Granny lineage (three carriers), the winter cultivars Yunona (two) and Yuka (one), and CIMMYT germplasm reached by two independent routes (line No. 153 and Roxo). No carrier derives the allele from indigenous Russian spring wheat breeding: of the three carriers bred from Russian crosses, CVT-87-23, CVT-89-23 and Lutescens 21/12-1-2, all obtained it from a winter parent, and the remaining carriers are introduced material or derived from it.

### 3.3. Plant Height, Lodging and Spike Traits in the Moscow Field Trial

Of the 43 accessions evaluated in both trials, three carried *Rht-B1b* (Granny, Granova and line No. 153), one carried *Rht-D1b* (Zolushka), and the remaining 39 carried the wild-type alleles at both loci. Plant height and spike traits were recorded on individual plants in the Moscow trial only; grain yield was determined in both trials and is reported in Section 3.4.

**Figure 3:**
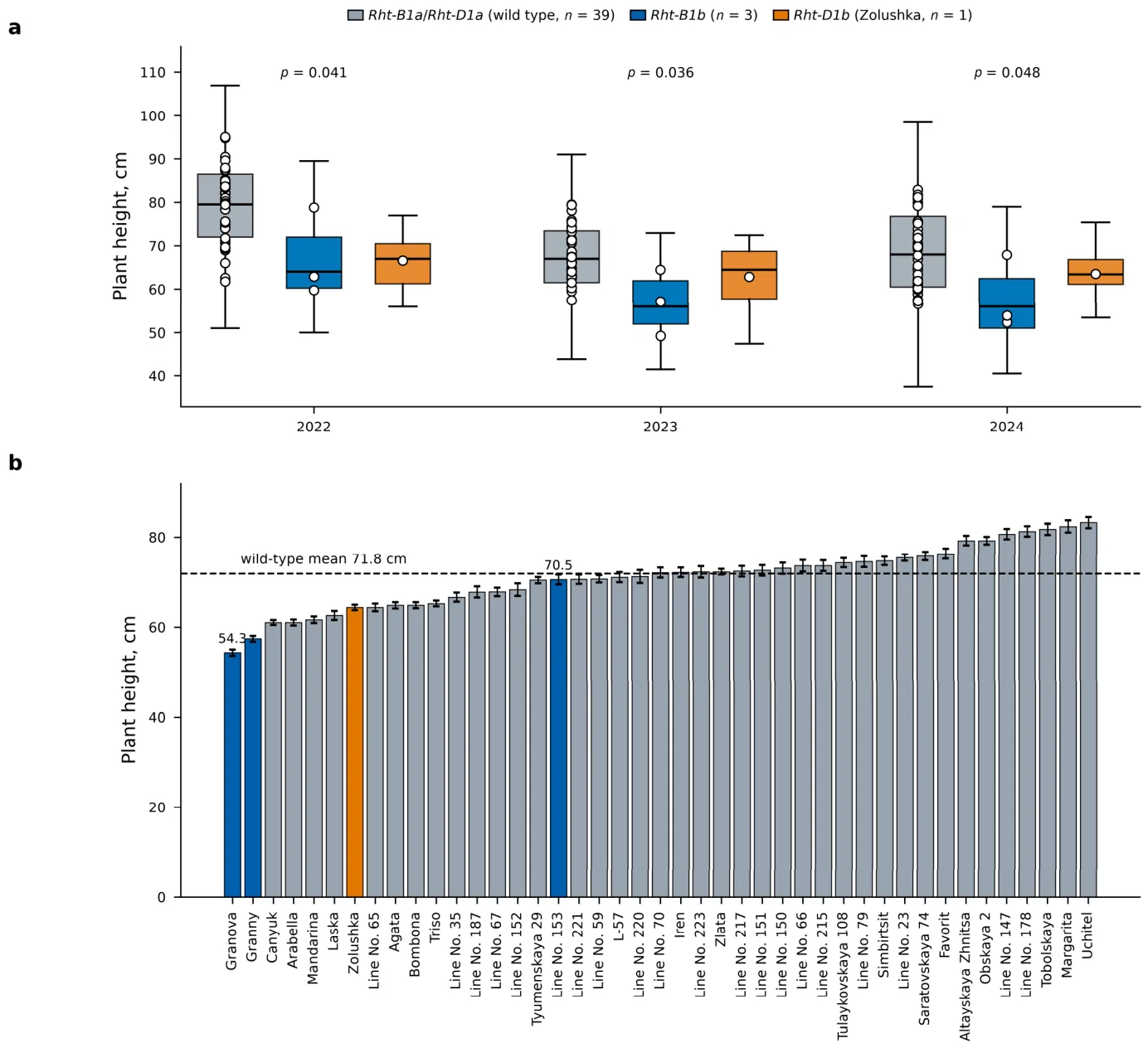
Plant height of spring bread wheat accessions with contrasting *Rht-B1* and *Rht-D1* allelic states, Moscow field trial, 2022–2024. (**a**) Distribution of individual plant height by year and allelic state. Each box summarises the individual plant records of all accessions in the group (approximately 30 plants per accession per year); boxes show the interquartile range and median, whiskers extend to 1.5 × IQR, and white circles indicate accession means. The boxes therefore display plant-level variation, whereas all significance tests were performed on accession means, as described in Section 2.4: the *Rht-B1b* group comprises 3 accessions, the wild-type group 39 and the *Rht-D1b* group a single accession (Zolushka), which was not tested; *p* values are from the two-sided Mann–Whitney U test on those means. (**b**) Mean plant height of individual accessions over the three seasons, ranked in ascending order; error bars indicate the standard error of the mean over individual plant records and the dashed line the mean of the 39 wild-type accessions.

Accessions carrying *Rht-B1b* were shorter than wild-type accessions in all three seasons (Table 2, Figure 3). Averaged over 2022–2024, mean plant height was 60.7 cm in the three *Rht-B1b* carriers and 71.8 cm in the 39 wild-type accessions, a reduction of 11.1 cm, or 15.4% (Mann–Whitney *U* test on accession means, *n* = 3 versus *n* = 39, *p* = 0.021). The reduction was 15.3% in 2022, 15.7% in 2023 and 15.5% in 2024 (*p* = 0.041, 0.036 and 0.048, respectively); with only three carriers, these *p* values indicate consistency across seasons, not precise estimates. The single *Rht-D1b* carrier, Zolushka, was 64.3 cm tall, 10.5% below the wild-type mean; this value is reported descriptively only. The height of the standard plots varied along the field (Zlata, from 73.2 to 88.0 cm in 2022, from 56.2 to 74.9 cm in 2023 and from 53.1 to 81.2 cm in 2024). After adjustment for field position with the repeated standard (Section 2.4), the carriers remained 14.6% shorter than the wild-type accessions (61.3 versus 71.8 cm, *p* = 0.018), with reductions of 15.1%, 14.8% and 13.8% in the three seasons (Supplementary Table S5). The adjusted height of Zolushka was 69.1 cm, 3.8% below the wild-type mean, so most of its apparent reduction reflected its position in a part of the field where the standard was also short.

Lodging occurred only sporadically in the Moscow trial. Mean lodging resistance across all 43 accessions was 4.86 in 2022, 4.49 in 2023 and 5.00 in 2024, and only four accessions fell below 4.5 as a three-year mean: Margarita (3.83), Uchitel (4.17), Tyumenskaya 29 and line No. 147 (4.33 each). All four carry the wild-type alleles, and two of them were the tallest accessions of the set (Uchitel 83.2 cm, Margarita 82.3 cm). Accessions carrying *Rht-B1b* scored 4.94 on average against 4.76 in wild-type accessions, and no difference was detected (*n* = 3 versus *n* = 39, *p* = 0.203). Across all 43 accessions, the three-year mean lodging score was nevertheless negatively correlated with plant height (Spearman ρ = −0.322, *p* = 0.035), so the scoring captured the expected relationship between stature and lodging even though the allelic groups could not be separated.

Reduced plant height was not accompanied by changes in spike traits. No differences were detected between the three *Rht-B1b* carriers and the 39 wild-type accessions in spike length (7.61 versus 7.50 cm, *p* = 0.712), the number of grains per spike (26.7 versus 27.3, *p* = 1.000) or grain weight per spike (1.21 versus 1.15 g, *p* = 0.371). Thousand-grain weight tended to be higher in the carriers (43.3 versus 41.1 g), although the difference was not significant (*p* = 0.124). The dwarfing allele shortened the culm without a detectable effect on spike productivity, although with three carriers the power to detect such an effect was low. Individual accession means revealed considerable variation within the *Rht-B1b* group (Figure 3b). Granova (54.3 cm) and Granny (57.4 cm) were the two shortest accessions of the entire set, whereas line No. 153, which carries the same allele, reached 70.5 cm and did not differ from the wild-type mean. Its height also remained within the wild-type range after adjustment for field position (69.5 cm). The marker result for this line agrees with its pedigree, which includes the *Rht-B1b* carrier Kauz (Section 3.2), so in this accession the allele was not accompanied by the reduction in height seen in Granny and Granova. The cause was not investigated; height-increasing alleles at other loci in its CIMMYT-derived background are one possibility, and such loci were not genotyped (Section 4.7).

### 3.4. Grain Yield in Contrasting Environments

**Figure 4:**
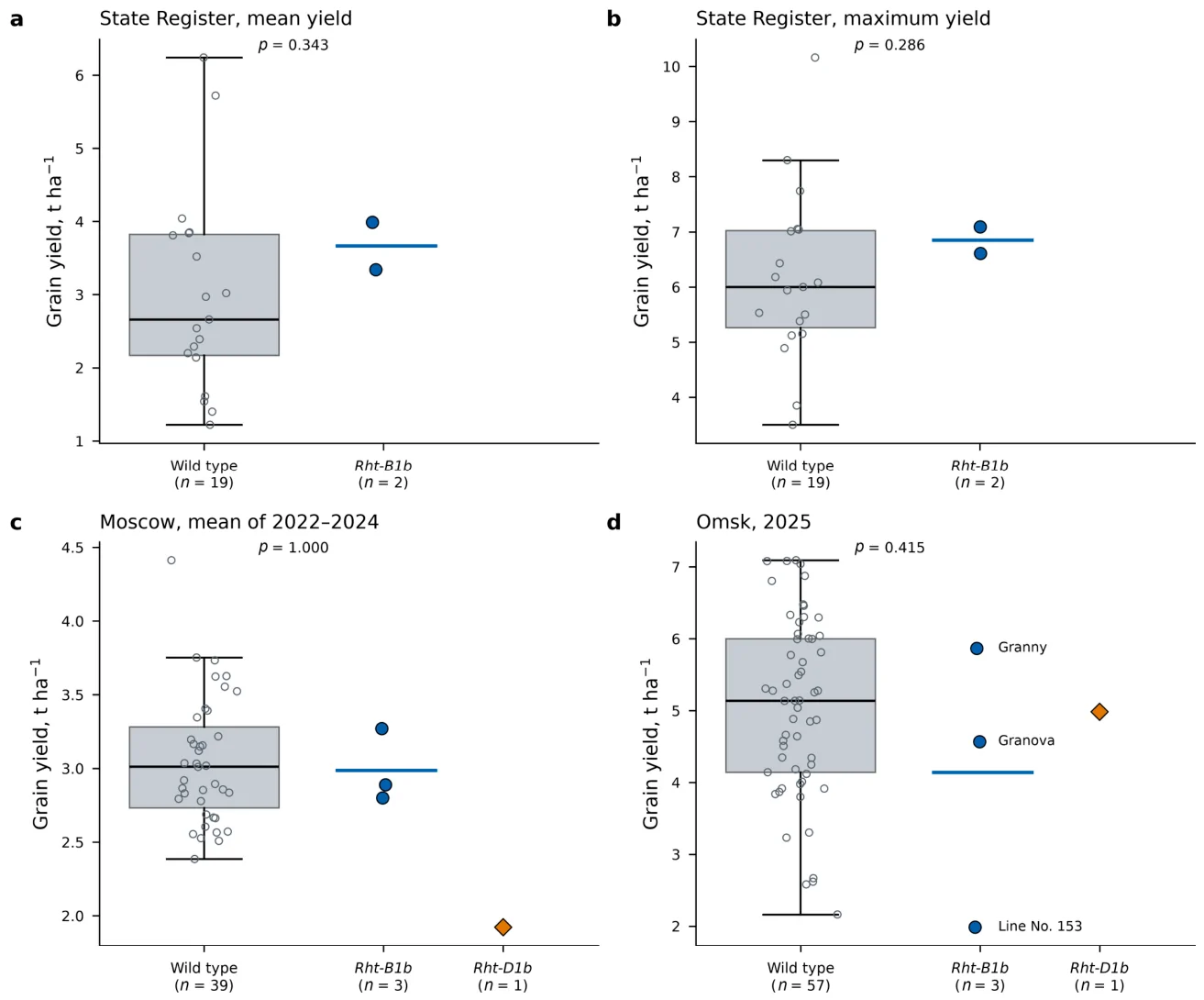
Grain yield of spring bread wheat accessions with contrasting alleles at the *Rht-B1* locus in different environments. (**a**,**b**) Mean and maximum yield of registered cultivars according to the State Register of Breeding Achievements. (**c**) Mean yield over three seasons in the Moscow field trial (Central Non-Chernozem zone). (**d**) Yield in the Omsk trial in 2025 (Western Siberia). Boxes show the interquartile range and median for the wild-type accessions (*Rht-B1a*/*Rht-D1a*), whiskers extend to 1.5 × IQR, and open circles denote individual accessions; accessions carrying *Rht-B1b* are shown as filled circles with their mean indicated by a horizontal line, and the single *Rht-D1b* carrier Zolushka, a breeding line with no State Register data, as a filled diamond in (**c**,**d**). *p* values are from the Mann–Whitney *U* test comparing *Rht-B1b* carriers with the wild-type accessions; Zolushka is excluded from both tested groups throughout. The number of accessions differs between panels because of data availability, and the Omsk trial was conducted in a single season; comparisons are descriptive and were not made across panels.

Grain yield did not follow plant height in either environment (Table 2, Figure 4). In the Moscow trial the three *Rht-B1b* carriers yielded 2.99 t ha^−1^ on average over 2022–2024 against 3.05 t ha^−1^ in the 39 wild-type accessions (*p* = 1.000), whereas season had a far stronger effect: mean yield was 4.30 t ha^−1^ in 2022 against 2.32 and 2.43 t ha^−1^ in 2023 and 2024. In the Omsk trial of 2025 (61 accessions, all genotyped here), the carriers averaged 4.14 t ha^−1^ against 5.05 t ha^−1^ in the 57 wild-type accessions (*p* = 0.415); this mean was driven by line No. 153, the lowest-yielding accession of the trial (1.98 t ha^−1^), whereas Granny (5.87 t ha^−1^) and Granova (4.57 t ha^−1^) fell within the wild-type range. Zolushka was the lowest-yielding accession in Moscow (1.92 t ha^−1^) but close to the trial mean in Omsk (4.98 t ha^−1^). State Register yields, which come from official variety testing and not from the present study, showed the opposite tendency: 3.67 t ha^−1^ for the two registered carriers against 3.00 t ha^−1^ for 19 wild-type cultivars, and maximum yields of 6.85 and 6.15 t ha^−1^ (*p* = 0.343 and *p* = 0.286, respectively). Protein content, gluten content and vitreousness did not differ between the allelic groups in either trial (Supplementary Tables S3, S3b and S5). Grain ash content was higher in the carriers in Omsk (3.36% against 2.47%, *p* = 0.033), but only because line No. 153 had the highest ash content of the trial (4.85%), Granny and Granova lying close to the trial mean. The mean overall breeding score assigned by the Omsk breeders was 4.67 for the three carriers (5.0 for Granny and Granova and 4.0 for line No. 153) against 3.65 for the 57 wild-type accessions, whose individual scores ranged from 2.0 to 5.0 (*p* = 0.038); five of the wild-type accessions also received the maximum score of 5.0. Confidence intervals and detection limits for all comparisons are given in Supplementary Table S5. Among the cultivars of the collection that appear in the annual rankings of the ten most widely sown spring wheat cultivars in the Russian Federation for 2020–2025, only Granny carries a dwarfing allele (Table 3). Three of the four carriers in the Moscow set, Granny, line No. 153 and Zolushka, were entered into the crossing programme (Table 4).

## 4. Discussion

### 4.1. Limited Occurrence of Rht-B1b and Rht-D1b in Spring Bread Wheat Germplasm

The classical gibberellin-insensitive dwarfing alleles were poorly represented in the studied spring bread wheat germplasm, with *Rht-B1b* detected in 2.2% and *Rht-D1b* in 0.3% of 370 accessions. *Rht-B1b* and *Rht-D1b* are among the most widely used dwarfing alleles in global wheat breeding, and they were central to the increase in lodging resistance and harvest index during the Green Revolution [1,2,4,5], so their rarity in the spring bread wheat examined here is unexpected.

Published surveys place their frequency an order of magnitude higher in other germplasm pools (Table 5). More than 90% of modern winter wheat cultivars of the central and eastern United States carry one of the two alleles [42], *Rht-D1b* and *Rht-B1b* occur at 45.5% and 24.5% in autumn-sown Chinese wheat [43], and *Rht-B1b* is effectively fixed in CIMMYT elite spring wheat [37]; comparable surveys have covered landraces and improved cultivars from Pakistan [44]. The closest comparison is Russian winter bread wheat genotyped by our group with the same assays: one of the three gibberellin-insensitive alleles occurred in 71% of 195 accessions grown at Krasnodar, *Rht-B1b* alone in 47% [20], and the *Rht-B1* locus has been surveyed in other Russian and Belarusian winter wheat collections [45,46]. *Rht-B1b* is therefore about twenty-fold rarer in the spring germplasm examined here, a difference that methodology cannot explain. Spring growth habit as such is not the cause, since *Rht-B1b* reached 69.6% in 199 spring wheat cultivars from Pakistan [47]. North American hard red spring wheat offers a partial parallel: *Rht-B1b* is now present in more than 65% of recent lines, but in parts of the Northern Plains adoption of both alleles remained low, breeders having relied on the gibberellin-sensitive *Rht24* and *Rht25*, and in the South Dakota programme only 20% of cultivars released after 1970 carried either allele [48].

The limited occurrence of these alleles should not be interpreted as evidence of their low breeding value as such. It may rather reflect the specific ecological and breeding conditions under which spring bread wheat has been selected in Russia. In many spring-wheat-growing regions, particularly in continental and drought-prone environments, breeding priorities extend beyond reduced plant height and lodging resistance to early vigour, sufficient vegetative biomass, drought adaptation and yield stability [49,50,51,52,53]. Under such conditions, the agronomic advantage of the Green Revolution alleles is not consistently realised: in trials conducted in Kazakhstan, China and South Africa, semi-dwarf genotypes carrying *Rht* alleles failed to outyield taller genotypes under drought [54], and near-isogenic comparisons in low-yielding dry environments of the western United States gave yield reductions of 9.6% at Creston, Montana and 12.3% at Horse Heaven, Washington for semi-dwarf *Rht-B1b* material [55]. In a Colorado RIL population evaluated across a range of soil moisture levels, lines combining both dwarfing alleles were significantly shorter, lower yielding and later heading in all environments, whereas lines carrying a single dwarfing allele did not outyield the tall class, apart from a marginal advantage of the *Rht-B1b* class in the highest-yielding environment [36].

The pedigrees point the same way: the allele appears to have entered Russian spring bread wheat through introduction or through the winter wheat gene pool and to have been propagated within a few lineages rather than dispersed across the breeding pool (Section 3.2).

### 4.2. Ecological and Breeding Factors Limiting the Use of Green Revolution Alleles

Spring bread wheat in Russia is cultivated across highly contrasting agroecological zones that differ in moisture availability, soil fertility, duration of the growing season, drought frequency and the level of agronomic input [50,51,52,53]. Where breeding has traditionally emphasised adaptation to unstable moisture, soil drought and yield stability, excessively reduced plant height or reduced early vigour may be undesirable, especially when selection is conducted under moderate or low-input backgrounds. Semi-dwarf genotypes carrying *Rht-B1b* or *Rht-D1b* may therefore be less competitive at early selection stages if they show reduced biomass accumulation, weaker seedling development or lower adaptation to stress [12,13,18].

A further mechanism links these alleles to establishment under dry conditions. Because *Rht-B1b* and *Rht-D1b* act through impaired gibberellin signalling, they shorten the coleoptile as well as the culm, whereas gibberellin-sensitive dwarfing genes such as *Rht18* reduce height alone [14,56]. In a genome-wide association analysis of coleoptile length in 150 bread wheat genotypes, the wild-type alleles *Rht-B1a* and *Rht-D1a* were associated with longer coleoptiles, and a marker on chromosome 4B lost its significance once the dwarfing genes were accounted for [57]. When the seedbed dries rapidly after sowing, seed is placed deeper, and genotypes with a short coleoptile emerge poorly, so that stand establishment rather than lodging limits yield [54,58]; semi-dwarf spring wheat grown under dry conditions has repeatedly shown reduced establishment from depth and lower water-use efficiency than taller genotypes [59,60,61,62]. Selection under such conditions acts against semi-dwarf forms before any advantage of reduced height can be expressed.

This may partly explain why the Green Revolution alleles, despite their global importance, remain rare in spring bread wheat. Their breeding value is realised most effectively under intensive agronomic conditions, where sufficient nitrogen input and favourable moisture increase the risk of lodging [3,10,11], whereas in drought-prone or low-input environments they may require genetic backgrounds that compensate for a shorter coleoptile, weaker early vigour and lower vegetative biomass [15,16,17].

### 4.3. Phenotypic Expression and Its Dissociation from Yield

The field evaluation indicated that *Rht-B1b* is phenotypically expressed in the studied germplasm, reducing plant height by about 15% consistently across three contrasting seasons. This magnitude is close to published estimates for single gibberellin-insensitive dwarfing alleles, for example a mean height reduction of 14.8% (11.4–17.8% across environments) for single dwarfing alleles in a spring wheat RIL population evaluated in four Colorado environments [36], and a mean reduction of about 12% attributable to *Rht-B1b* or *Rht-D1b* across the North American hard red spring wheat region, falling to about 9% in its dry environments [48]. The reduction observed here (15.4%) lies within the Colorado range and exceeds the North American regional mean, which is consistent with the more favourable moisture supply of the Central Non-Chernozem zone; its stability across seasons also supports the marker-based classification.

Reduced height was not accompanied by higher yield in either trial. In Moscow, where no nitrogen was applied beyond the pre-sowing dose, lodging pressure was minimal: in 2024 every accession received the maximum score, and across the three seasons only four accessions, all wild-type and three of them among the five tallest, showed appreciable lodging, so the principal agronomic advantage of the Green Revolution alleles had little opportunity to be expressed. In Omsk the carriers averaged below the wild type, but this mean was driven by a single CIMMYT-derived line, which was also the lowest-yielding of the six genotypes in our separate six-season assessment in the Non-Chernozem zone [63]. A Nordic–Baltic panel of 299 spring wheat genotypes likewise showed no beneficial effect of *Rht-B1b* or *Rht-D1b* on grain yield across twelve environments, the carriers being generally low-yielding [64]. Grain quality did not differ between the allelic groups in either environment (Section 3.4). The Omsk breeding score is a subjective assessment that takes plant height and lodging into account and is therefore not independent of the trait under study, but the two *Rht-B1b* semi-dwarfs were among the seven of 61 accessions that received the maximum score, so breeders in the target region rated this material highly. The effect of the alleles also depends on the production system and the genetic background: the relative performance of gibberellin-responsive and gibberellin-insensitive alleles differed between tillage systems [65], near-isogenic spring wheat lines differing in major reduced-height genes varied in adaptation across international environments [66], and in bread wheat of southern Ukraine the expression of dwarfing genes depended on the cultivar background [67].

The contrast between Granny and Granova, the two shortest accessions of the whole set, and line No. 153, which carries the same allele but reached wild-type height, is consistent with a dependence of *Rht-B1b* expression on the genetic background, although with a single such line this cannot be distinguished from other causes.

### 4.4. Donor Accessions Carrying Rare Dwarfing Alleles

The carriers identified here are rare donors of the classical Green Revolution alleles in spring bread wheat, and Granny and Granova are of the most immediate breeding interest. Granny is a widely used spring bread wheat cultivar which, according to the annual rankings of the Russian Agricultural Center, has been among the ten leading spring wheat cultivars by seed volume sown in the Russian Federation throughout 2020–2025, and in 2025 its seed volume of 106.1 thousand tonnes was the largest among the cultivars of the present collection that appear in those rankings (Table 3) [38,39,40,41]. Granova was described in the State Register as originating from individual selection from Granny [24]. The presence of *Rht-B1b* in both cultivars is consistent with their pedigree relationship and shows that the allele can be retained and transmitted in spring wheat breeding. Granny carries, in addition to *Rht-B1b*, the powdery mildew resistance gene *Pm3d* and the leaf rust resistance gene *Lr21*, the latter introgressed from *Aegilops tauschii* [68,69]. It is thus a donor of several agronomically valuable genes simultaneously, although its bread-making quality is rated only as that of a good filler wheat. Granny is also the only carrier of *Rht-B1b* among the leading cultivars included in the present collection: Likamero, Iren, KWS Buran, Omskaya 36, KWS Torridon, Triso and Uralosibirskaya all carry the wild-type alleles at both loci. Likamero and KWS Buran, which carry the wild-type alleles, are nevertheless described in the State Register, like Granny, as resistant to lodging [24], so lodging resistance in this germplasm has been achieved without *Rht-B1b* or *Rht-D1b*.

The detection of *Rht-D1b* in Zolushka is of particular interest, since this accession is the only source of the *Rht2* allele in the collection. The presence of a target dwarfing allele alone, however, does not indicate breeding value. Zolushka combines several traits that limit its direct use: blue aleurone, a brittle rachis and low productivity, a combination more characteristic of primitive than of modern bread wheat, which complicates both harvesting and seed multiplication. It was the lowest-yielding accession in the Moscow trial (1.92 t ha^−1^), it gave the lowest yield of eleven accessions in a variety trial in the Orel region [33], and it showed high susceptibility to powdery mildew in our earlier assessments [68]. Zolushka is therefore of value only as a donor of *Rht-D1b*, in crosses followed by selection against these traits.

### 4.5. Winter Wheat as a Contrasting Example

Russian winter bread wheat provides a useful contrast. In winter wheat breeding, especially in southern programmes focused on high-yielding cultivars for intensive production, reduced-height genes have been widely incorporated into breeding material [19,20,21,70], and *Rht-B1b* is about twenty times more frequent in Russian winter bread wheat than in the spring germplasm analysed here (Section 4.1; Table 5). The role of reduced-height genes in the Krasnodar winter wheat breeding school has been reviewed in this context [71].

This contrast suggests that the incorporation of dwarfing alleles depends strongly on crop type, target environment and selection strategy. Winter wheat cultivars developed for intensive systems benefit more consistently from reduced plant height, because high nitrogen input and favourable moisture increase lodging risk, so that dwarfing alleles contribute directly to plant architecture and yield potential [1,3,10,19]. In spring bread wheat, especially in continental regions with drought stress and unstable moisture, selection may instead favour genotypes with stronger early development, greater plant robustness and stable yield formation under stress [50,51,52,53].

The two crops are not, however, entirely different: three of the eight carriers inherited *Rht-B1b* from a winter bread wheat parent, at Nemchinovka and at Omsk independently (Section 3.2). Winter wheat may therefore serve as an accessible donor pool for spring wheat improvement, provided that the introgressed segments are evaluated under spring wheat growing conditions.

### 4.6. Implications for Marker-Assisted Breeding

Because *Rht-B1b* and *Rht-D1b* occur at very low frequencies and are not always expressed in plant height, their carriers can go unrecognised in conventional phenotypic selection. In the material examined here, line No. 153 carries *Rht-B1b* yet did not differ in height from wild-type accessions, the breeding line Lutescens 21/12-1-2 carries the allele while reaching 83 cm [35], and the two Nemchinovka carriers reached 80 cm [23]. None of them would readily be recognised as a carrier by phenotypic inspection. Molecular markers make it possible to identify and retain such genotypes intentionally, even when the phenotypic effect is not expressed in a given genetic background [4,22,25,53,72].

Marker-assisted selection should therefore aim not at raising the frequency of these alleles in spring wheat but at introducing them into appropriate genetic backgrounds and evaluating them under the target conditions. Under intensive management on the better-supplied soils of the forest-steppe, where lodging can limit yield, semi-dwarf cultivars carrying *Rht-B1b* or *Rht-D1b* may be preferable [1,3,4,10]. In the dry steppe of Western Siberia and northern Kazakhstan, where lodging is seldom limiting, these alleles would need to be combined with a long coleoptile, strong early vigour and sufficient biomass [13,18,54,58], or gibberellin-sensitive dwarfing genes could be used instead in sesqui-dwarf combinations [73].

The rarity of *Rht-B1b* and *Rht-D1b* does not mean that the studied germplasm is uniformly tall. Among the 39 wild-type accessions, plant height ranged from 61.0 cm (Canyuk and Arabella) to 83.2 cm (Uchitel), and four of them were shorter than the *Rht-D1b* carrier (64.3 cm), so reduced height in this material is also conferred by other loci. Gibberellin-sensitive dwarfing genes are plausible candidates. *Rht8* reduces plant height by about 5–10 cm depending on the environment, without an adverse effect on yield in European trials [74,75], and has been assessed under contrasting nitrogen and water regimes [76]; the 192 bp allele of its marker *Xgwm261* is, however, not diagnostic in all pedigrees [77,78]. This marker allele was found in ten of fourteen Omsk breeding lines, including Lutescens 21/12-1-2 [35], and has been surveyed in the Moscow material examined here [63]. In the Non-Chernozem zone the gibberellin-responsive *Rht13* reduced plant height by 13.0 cm (17.4%) while increasing grain number per main spike and the number of productive tillers [79]. *Rht24*, a gibberellin 2-oxidase gene, reduces height without a yield penalty, and its dwarfing allele, which arose in wild emmer, occurs in more than half of the wild emmer and wheat accessions examined worldwide, so it may be present in spring wheat germplasm without deliberate introduction [80,81,82,83]. In North American spring wheat the dwarfing alleles at *Rht24* and *Rht25* have increased in frequency in recent decades and interact with *Rht-B1* and *Rht-D1* [48]. Identifying the loci responsible in the present collection was beyond the scope of this study.

Twenty-seven Rht loci have now been described, ten of them cloned, and *Rht24* was the most frequent in a recent survey of 47 varieties (91.5%) [84]. New loci continue to be reported, among them *Rht28* on chromosome arm 2AL [85] and the non-canonical gibberellin-sensitive *Rht29* [86]. Two further gibberellin-insensitive *Rht-B1* alleles are used in Russian breeding: *Rht-B1e* (=*Rht11*) [30,70] and *Rht-B1p* (=*Rht17*), for which an allele-specific marker has been developed [31] and whose effects have been characterised in spring bread wheat in the Central Non-Chernozem region and Krasnodar Krai [87]. Neither allele was found in the present collection (Section 3.1).

The carriers have already entered practical use. Granny, line No. 153 and Zolushka were used as donors in 26 cross combinations at the All-Russian Research Institute of Agricultural Biotechnology (Table 4). Nine combinations use cultivars released for the Urals and Western Siberia (Pamyati Tyunina, Uralosibirskaya, Tyumenskaya 29, Element 22 and Silantiy) as female parents, so that the dwarfing allele is introduced into locally adapted backgrounds, and the remaining combinations are directed at the Central Non-Chernozem zone. The fourteen spring wheat parents that belong to the present collection all carry the wild-type alleles at both loci, so in the corresponding combinations the donor is the only source of a Green Revolution dwarfing allele; the fifteenth parent, Silantiy, was not part of the genotyped collection. Backcrossing with Zolushka as a donor of *Rht-D1b* is under way within the Kazakhstan–Siberia Network on Spring Wheat Improvement (KASIB), and two combinations use winter bread wheat cultivars as pollen parents, the same route by which the dwarfing alleles reached the carriers described above.

### 4.7. Limitations

Several limitations should be borne in mind. The dwarfing alleles were represented by very few accessions (eight carriers of *Rht-B1b* and a single carrier of *Rht-D1b* among 370), so all comparisons involving allelic groups are necessarily exploratory. With three carriers against 39 wild-type accessions, and given the variation observed among the wild-type accessions, the smallest difference detectable with 80% power was 10.2 cm for plant height (14.1% of the wild-type mean), 0.73 t ha^−1^ for grain yield in Moscow (23.8%) and 0.21 g for grain weight per spike (18.0%). The observed height reduction of 11.1 cm only just exceeded the detection limit of the design. The yield comparison constrains the effect much less tightly: the 95% confidence interval for the difference ranged from −0.52 to +0.41 t ha^−1^ in Moscow and from −3.15 to +1.23 t ha^−1^ in Omsk. The absence of a significant yield difference should accordingly be read as excluding a change larger than about 0.5 t ha^−1^ in Moscow, and as essentially uninformative in Omsk, rather than as evidence that the allele is yield-neutral. For thousand-grain weight the interval (−0.72 to +5.24 g) leaves an agronomically appreciable advantage of the carriers open. All intervals and detection limits are given in Supplementary Table S5. The field evaluation covered 43 of the 370 accessions in Moscow and 61 in Omsk, and the two locations were tested in different years, so that location and season effects cannot be separated. The Omsk trial comprised a single season, and in both trials the small plots give only comparative yields. The Moscow trial followed a systematic design with a repeated standard, not a randomised one; adjustment for field position left the height difference between the allelic groups essentially unchanged (Section 3.3), but position effects on other traits cannot be excluded. Lodging was scored in the Moscow trial only and, because lodging pressure was low in all three seasons, the data do not allow the effect of the dwarfing alleles on lodging resistance to be assessed under conditions where lodging would be agronomically limiting. Pedigree assignments rest largely on documentary records; only for Lutescens 21/12-1-2 were both parents genotyped (Section 3.2). The two accessions heterogeneous at *Rht-B1* were each represented by only two individual plants and one bulk sample, so the frequency of the *Rht-B1b* fraction within them is unknown, and the discrepancy with an earlier survey of the same programme [23] suggests that the detection of rare within-accession variants depends on sample size. Finally, alleles at the two loci other than *Rht-B1b*, *Rht-B1e*, *Rht-B1p* and *Rht-D1b*, and other reduced-height loci, such as *Rht8* and *Rht24*, were not genotyped, so the loci responsible for reduced height in the wild-type accessions remain unidentified. The collection was assembled from the material accessible to the participating programmes and is not a probability sample of the spring bread wheat grown in Russia: almost half of the accessions originate from two institutions in the Omsk region, and the frequencies reported here therefore describe this collection rather than the national germplasm. No neutral marker data were generated for these accessions, so the genetic relationships among them and the diversity represented in the collection could not be assessed.

## 5. Conclusions

Of the 370 spring bread wheat accessions examined, eight carried *Rht-B1b* (2.2%) and one carried *Rht-D1b* (0.3%); these frequencies describe the collection studied rather than Russian spring bread wheat as a whole. The eight *Rht-B1b* carriers trace to five sources, and none derives the allele from indigenous Russian spring wheat breeding: three inherited it from a winter bread wheat parent, two from CIMMYT germplasm, and three belong to a single introduced lineage. In the 43 accessions evaluated in Moscow, the carriers were 15.4% shorter than the wild-type accessions, with no detectable difference in spike traits or in grain yield in Moscow or Omsk; with three carriers, these comparisons are exploratory. Because one of the three field-evaluated carriers did not differ in height from the wild-type accessions, marker-based identification is needed wherever these donors are deployed. Three directions follow from these results. First, the collection was genotyped at *Rht-B1* and *Rht-D1* only, yet several wild-type accessions were shorter than the single *Rht-D1b* carrier; genotyping at the gibberellin-sensitive loci, in particular *Rht8*, *Rht24* and *Rht25*, would identify the loci that confer reduced height in this germplasm and show which of them Russian spring wheat breeding has used in place of the Green Revolution alleles. Second, characterising the collection with neutral markers would describe its genetic structure and place the frequency estimates reported here in a wider context. Third, the carriers are now used as donors in the crossing and backcrossing programmes described above, and the populations derived from them will allow the effects of the alleles to be estimated in adapted backgrounds and across the contrasting environments of the Central Non-Chernozem zone and Western Siberia, where coleoptile length and emergence from deeper sowing matter as much as the reduction in height itself.

## Figures and Tables

**Table 1:** Breeding origin of the accessions carrying dwarfing alleles or showing a heterogeneous state at the *Rht-B1* locus.

Accession	Allele	Pedigree	Inferred Donor	Evidence ^1^
Granny	*Rht-B1b*	U23 × U513	U513; growth habit not stated in the registration document	Pedigree only
Granova	*Rht-B1b*	Individual selection from Granny	Granny	Pedigree + genotype
Pamyati Konovalova	*Rht-B1b*	(Laval 19 × Granny) × Granny	Granny	Pedigree only
CVT-87-23	*Rht-B1b*	Line 1962-21 (h3439) [Dandy × line 5662-13 (h2823) (Yunona × Zlata) F8] F6	Yunona (winter)	Pedigree + published genotype
CVT-89-23	*Rht-B1b*	Line 1993-21 (h3444) [Kolo (winter) × line 5662-13 (h2823) (Yunona × Zlata) F8] F6	Yunona or Kolo, both winter	Pedigree + published genotype
Lutescens 21/12-1-2	*Rht-B1b*	Omskaya 36 × Yuka	Yuka (winter)	Both parents verified
Line No. 153	*Rht-B1b*	Lutescens 1085/7/Tob/Era//Tob/Cno67/3/Plo/4/Vee#5/5/Kauz/6/Fret2	CIMMYT semi-dwarf ancestry; not assignable to a single parent	Pedigree only
Roxo	*Rht-B1b*	VS73.600/Mirlo/3/Bobwhite//Yécora 70/Trifon “S”	CIMMYT semi-dwarf ancestry; not assignable to a single parent	Pedigree only
CVT-102-23	*Rht-B1a/Rht-B1b (het.)*	Agata × line 4551-14 (Kurier × Liza) F7	Unresolved; Kurier not genotyped	Pedigree only
CVT-123-23	*Rht-B1a/Rht-B1b (het.)*	Line 5412-21 (h3515) [line 4321-14 (h2984) (Ester × Liza) × Moskovka] F6	Unresolved; Moskovka not genotyped	Pedigree only
Zolushka	*Rht-D1b*	Not available	Unknown	No pedigree record

^1^ Note: Evidence is classified as follows. “Both parents verified” indicates that the donor genotype has been published and the second parent was genotyped in this study; “Pedigree + published genotype”, that the genotype of a parent or ancestor has been published but the second parent was not genotyped; “Pedigree + genotype”, that parent and derivative were both genotyped here; “Pedigree only”, that the assignment rests on documentary records alone. CVT—competitive variety trial; het.—heterogeneous accession, in which both *Rht-B1a* and *Rht-B1b* were detected among the plants analysed (Section 3.2).

**Table 2:** Allelic state at the *Rht-B1* and *Rht-D1* loci and agronomic traits of the 43 spring bread wheat accessions evaluated in the field. Plant height and spike traits are means of individual plant records from the trial at the Field Experiment Station of the Russian State Agrarian University—Moscow Timiryazev Agricultural Academy (Moscow, Russia) for 2022–2024; grain yield is given for the same trial and for the trial in the Omsk region (Western Siberia) in 2025. Accessions carrying a dwarfing allele are highlighted.

No.	Accession	Allelic State	Plant Height, cm	Spike Length, cm	Grains per Spike	Grain Weight per Spike, g	TGW, g	Grain Yield Moscow, t ha^−1^	Grain Yield Omsk, t ha^−1^
1	Zlata	*Rht-B1a*/*Rht-D1a*	72.3	7.45	24.7	1.02	41.8	3.35	5.13
2	Saratovskaya 74	*Rht-B1a*/*Rht-D1a*	75.8	7.10	26.2	1.17	43.2	3.16	2.62
3	Agata	*Rht-B1a*/*Rht-D1a*	64.8	7.50	31.1	1.23	39.1	2.92	4.35
4	Tulaykovskaya 108	*Rht-B1a*/*Rht-D1a*	74.3	7.65	26.9	1.15	42.3	3.01	3.84
5	Simbirtsit	*Rht-B1a*/*Rht-D1a*	74.7	7.20	24.8	1.10	45.0	3.55	5.37
6	Tyumenskaya 29	*Rht-B1a*/*Rht-D1a*	70.4	6.95	26.4	1.07	40.0	3.15	5.81
7	Obskaya 2	*Rht-B1a*/*Rht-D1a*	79.2	7.41	27.0	1.28	45.6	3.75	3.92
8	Tobolskaya	*Rht-B1a*/*Rht-D1a*	81.7	7.27	25.8	1.10	41.6	3.62	2.67
9	Altayskaya Zhnitsa	*Rht-B1a*/*Rht-D1a*	79.2	8.28	25.6	1.13	43.4	3.41	3.80
10	Margarita	*Rht-B1a*/*Rht-D1a*	82.3	7.54	28.0	1.31	46.2	4.41	5.49
11	Uchitel	*Rht-B1a*/*Rht-D1a*	83.2	7.63	27.4	1.22	41.9	2.83	3.91
12	Favorit	*Rht-B1a*/*Rht-D1a*	76.3	7.95	28.0	1.12	36.4	3.02	5.14
13	Granny	*Rht-B1b*/*Rht-D1a*	57.4	7.51	28.2	1.24	42.8	3.27	5.87
14	Triso	*Rht-B1a*/*Rht-D1a*	65.2	6.91	26.6	1.08	38.8	3.12	5.67
15	Iren	*Rht-B1a*/*Rht-D1a*	72.1	8.10	25.6	1.03	38.7	3.16	5.28
16	Bombona	*Rht-B1a*/*Rht-D1a*	64.8	7.47	30.2	1.20	38.4	2.79	3.98
17	L-57	*Rht-B1a*/*Rht-D1a*	71.0	8.64	30.4	1.07	36.6	2.57	5.28
18	Laska	*Rht-B1a*/*Rht-D1a*	62.6	7.58	30.9	1.24	39.6	3.52	3.87
19	Arabella	*Rht-B1a*/*Rht-D1a*	61.0	6.05	25.6	1.01	39.4	3.63	6.04
20	Granova	*Rht-B1b*/*Rht-D1a*	54.3	7.50	24.5	1.09	42.8	2.89	4.57
21	Mandarina	*Rht-B1a*/*Rht-D1a*	61.6	6.63	33.0	1.22	37.2	3.73	5.77
22	Zolushka	*Rht-B1a*/*Rht-D1b*	64.3	7.50	23.3	0.78	33.2	1.92	4.98
23	Canyuk	*Rht-B1a*/*Rht-D1a*	61.0	6.56	26.9	1.17	43.8	3.22	7.04
24	Line No. 23	*Rht-B1a*/*Rht-D1a*	75.4	7.88	26.6	1.18	42.8	3.03	6.00
25	Line No. 35	*Rht-B1a*/*Rht-D1a*	66.6	7.84	27.5	1.28	46.2	2.69	3.30
26	Line No. 59	*Rht-B1a*/*Rht-D1a*	70.7	7.31	22.7	0.89	40.3	2.83	4.51
27	Line No. 65	*Rht-B1a*/*Rht-D1a*	64.3	7.57	18.4	0.92	44.2	2.39	6.23
28	Line No. 66	*Rht-B1a*/*Rht-D1a*	73.6	6.93	26.6	1.12	41.0	2.78	5.54
29	Line No. 67	*Rht-B1a*/*Rht-D1a*	67.8	6.65	26.0	1.04	40.0	2.51	4.58
30	Line No. 70	*Rht-B1a*/*Rht-D1a*	72.1	7.16	30.3	1.28	39.6	2.86	6.87
31	Line No. 79	*Rht-B1a*/*Rht-D1a*	74.5	7.94	31.5	1.28	40.8	2.87	3.23
32	Line No. 147	*Rht-B1a*/*Rht-D1a*	80.6	8.66	30.0	1.24	40.6	3.03	7.08
33	Line No. 150	*Rht-B1a*/*Rht-D1a*	73.0	8.00	27.1	1.06	39.2	2.60	4.18
34	Line No. 151	*Rht-B1a*/*Rht-D1a*	72.6	7.47	24.3	1.06	42.9	2.67	4.66
35	Line No. 152	*Rht-B1a*/*Rht-D1a*	68.3	6.79	25.8	1.09	41.5	2.53	6.48
36	Line No. 153	*Rht-B1b*/*Rht-D1a*	70.5	7.81	27.6	1.30	44.3	2.80	1.98
37	Line No. 178	*Rht-B1a*/*Rht-D1a*	81.3	8.27	33.9	1.36	38.8	3.19	2.16
38	Line No. 187	*Rht-B1a*/*Rht-D1a*	67.8	7.49	31.2	1.45	42.0	2.66	7.08
39	Line No. 215	*Rht-B1a*/*Rht-D1a*	73.6	7.61	25.8	1.17	41.7	2.85	6.33
40	Line No. 217	*Rht-B1a*/*Rht-D1a*	72.4	7.68	23.9	1.14	48.1	2.89	6.30
41	Line No. 220	*Rht-B1a*/*Rht-D1a*	71.2	7.85	26.7	1.04	37.5	2.57	6.00
42	Line No. 221	*Rht-B1a*/*Rht-D1a*	70.6	7.35	24.0	0.93	37.2	2.55	5.04
43	Line No. 223	*Rht-B1a*/*Rht-D1a*	72.2	8.24	31.3	1.31	40.0	3.39	4.64

Note: TGW—thousand-grain weight. Values were recalculated from the primary records; ten of 4038 individual plant records with implausible values (plant height < 30 cm, spike length < 3 cm, or grain weight per spike > 5 g) were excluded as data-entry errors and are listed in Supplementary Table S6. LSD_0.05_ values for each year and trait are given in Supplementary Table S7.

**Table 3:** Seed volumes sown (thousand tonnes) of leading Russian spring wheat cultivars in 2020–2025 and their allelic state at the *Rht-B1* and *Rht-D1* loci. Data from the annual rankings of the Russian Agricultural Center [38,39,40,41], which cover spring wheat as a whole; only the bread wheat cultivars that are included in the present collection are shown. A dash indicates that the cultivar was not among the ten leading cultivars in that year.

Cultivar	*Rht-B1*	*Rht-D1*	2020	2021	2022	2023	2024	2025
Granny	*Rht-B1b*	*Rht-D1a*	64.7	102.7	79.5	115.9	110.1	106.1
Likamero	*Rht-B1a*	*Rht-D1a*	24.2	62.3	77.3	123.1	108.7	103.9
Iren	*Rht-B1a*	*Rht-D1a*	147.2	162.9	145.7	128.9	106.8	71.5
KWS Buran	*Rht-B1a*	*Rht-D1a*	45.4	16.4	45.5	71.7	80.1	61.2
Omskaya 36	*Rht-B1a*	*Rht-D1a*	137.9	114.6	93.7	77.0	53.7	—
KWS Torridon	*Rht-B1a*	*Rht-D1a*	—	—	35.5	63.7	49.5	52.2
Triso	*Rht-B1a*	*Rht-D1a*	65.6	58.3	42.3	52.1	—	—
Uralosibirskaya	*Rht-B1a*	*Rht-D1a*	70.2	62.3	51.9	—	—	—

**Table 4:** Cross combinations in which accessions carrying Green Revolution dwarfing alleles were used as parents. Crosses were made by hand emasculation and pollination under controlled conditions.

No.	Cross Combination (♀ × ♂)	Donor Allele	Generation Reached	Notes
1	Granny × Zlata	*Rht-B1b*	F2	reciprocal crosses made
2	Likamero × Granny	*Rht-B1b*	F2	
3	Granny × Yoldyz	*Rht-B1b*	F1	
4	Granny × Liza	*Rht-B1b*	F1	reciprocal crosses made
5	Pamyati Tyunina × Granny	*Rht-B1b*	F1	
6	Line No. 35 × Granny	*Rht-B1b*	F1	
7	Granny × Ermolovka	*Rht-B1b*	F1	winter pollen parent
8	Granny × Zyuganovka	*Rht-B1b*	F2	winter pollen parent
9	Zlata × Line No. 153	*Rht-B1b*	F1	
10	Iren × Line No. 153	*Rht-B1b*	F1	
11	Line No. 153 × Odeta	*Rht-B1b*	F2	
12	Zlata × Zolushka	*Rht-D1b*	F2	
13	Yoldyz × Zolushka	*Rht-D1b*	F1	
14	Alvaris × Zolushka	*Rht-D1b*	F1	
15	Odeta × Zolushka	*Rht-D1b*	F1	reciprocal crosses made
16	Arabella × Zolushka	*Rht-D1b*	F1	reciprocal crosses made
17	Line No. 35 × Zolushka	*Rht-D1b*	F1	
18	Silantiy × Zolushka	*Rht-D1b*	F1	
19	Pamyati Tyunina × Zolushka	*Rht-D1b*	F1	
20	Uralosibirskaya × Granny	*Rht-B1b*	F1	
21	Uralosibirskaya × Zolushka	*Rht-D1b*	F1	
22	Tyumenskaya 29 × Granny	*Rht-B1b*	F1	
23	Tyumenskaya 29 × Zolushka	*Rht-D1b*	F1	
24	Element 22 × Granny	*Rht-B1b*	F1	
25	Element 22 × Zolushka	*Rht-D1b*	F1	
26	Canyuk × Zolushka	*Rht-D1b*	F1	

Note: Combinations involving Zolushka, the only carrier of *Rht-D1b*, are shaded. The female parent is given first. Combinations for which the reciprocal cross was also made are marked in the Notes column. Generation reached indicates the most advanced generation obtained to date. Two combinations used winter bread wheat cultivars as pollen parents.

**Table 5:** Reported frequencies of the Green Revolution dwarfing alleles *Rht-B1b* and *Rht-D1b* in different bread wheat germplasm pools.

Source	Germplasm	*n*	*Rht-B1b*, %	*Rht-D1b*, %	Marker System
Present study	Spring bread wheat, diverse origin, mainly Russia	370	2.2	0.3	KASP
[23]	Spring bread wheat, FRC “Nemchinovka”	71	2.8	0	KASP
[20]	Winter bread wheat, Krasnodar, Russia	195	47	14	KASP
[42]	Winter bread wheat, central and eastern USA	362	n.d.	45	Allele-specific PCR
[43]	Autumn-sown bread wheat, China	220	24.5	45.5	Allele-specific PCR
[37]	Elite spring bread wheat, CIMMYT	369	~100	Rare	Functional markers
[47]	Spring bread wheat, Pakistan	199	69.6	n.d.	KASP
[48]	Hard red spring wheat, North America	1013	>65 (last two decades)	<10 (last two decades)	SNP array, KASP

Note: n.d., not determined. The studies differ in germplasm definition, sample size and marker system, so the values are given for comparison only and no pooled estimate is implied. *Rht-B1e* was also detected in 9% of the winter wheat accessions [20], and 29% of them carried none of the three alleles. The accessions surveyed in [23] include the two carriers identified here (CVT-87-23 and CVT-89-23), so that estimate is not independent of the present one.

## Data Availability

All data supporting the findings of this study are available within the article and its Supplementary Materials. The primary individual-plant records from the Moscow field trial are available from the corresponding author on reasonable request.

## Supplementary Materials

The following supporting information can be downloaded at: https://www.mdpi.com/article/10.3390/plants15213270/s1, Table S1, plant material and pedigrees of the 370 accessions; Table S2, allelic states at *Rht-B1* and *Rht-D1* for the 370 accessions; Table S3, grain yield of the field-evaluated accessions in Moscow and Omsk, State Register yield of the registered cultivars, lodging resistance scores, and grain protein, gluten and vitreousness for the Moscow trial; Table S3b, grain yield of all 61 accessions of the Omsk trial with grain quality, vegetation period and overall breeding score; Table S4, mean daily air temperature and total precipitation during the growing season in Moscow, 2022–2024; Table S5, Hodges–Lehmann estimates, 95% confidence intervals and detection limits for the comparisons between allelic groups, including plant height adjusted for field position, lodging resistance and the grain quality traits of the Omsk trial; Table S6, the ten individual plant records excluded as implausible; Table S7, analysis of variance and LSD_0.05_ for the Moscow field trial by year and trait.

## Abbreviations

The following abbreviations are used in this manuscript:

CIMMYT	International Maize and Wheat Improvement Center
CTAB	Cetyltrimethylammonium bromide
CVT	Competitive variety trial
DNA	Deoxyribonucleic acid
IQR	Interquartile range
KASIB	Kazakhstan–Siberia Network on Spring Wheat Improvement
KASP	Kompetitive allele-specific PCR
LSD	Least significant difference
PCR	Polymerase chain reaction
RIL	Recombinant inbred line
SNP	Single nucleotide polymorphism
TGW	Thousand-grain weight
VIR	N.I. Vavilov All-Russian Institute of Plant Genetic Resources
Rht	Reduced height (wheat gene symbol)
